# The Effect of Physical Rehabilitation on Oro-Motor Stimulation, Manual Airway Clearance, Positioning, and Tactile Stimulation (PROMPT) on Neonates With Respiratory Distress Syndrome

**DOI:** 10.7759/cureus.64656

**Published:** 2024-07-16

**Authors:** H V Sharath, Moh'd Irshad Qureshi, Raghumahanti Raghuveer, Akshaya Saklecha, Pavan T Nadipena

**Affiliations:** 1 Department of Pediatric Physiotherapy, Ravi Nair Physiotherapy College, Datta Meghe Institute of Higher Education and Research, Wardha, IND; 2 Department of Neuro Physiotherapy, Ravi Nair Physiotherapy College, Datta Meghe Institute of Higher Education and Research, Wardha, IND; 3 Department of Osteopathy, Dr Hullumani's Polyclinic and Rehabilitation, Bangalore, IND

**Keywords:** neonatal respiratory distress syndrome, high-risk neonates, chest physiotherapy, physiotherapy, nrds

## Abstract

Neonatal respiratory distress syndrome (NRDS) is a significant cause of morbidity and mortality in preterm infants due to insufficient surfactant production in the lungs. This case report explores the effect of physical rehabilitation on oro-motor stimulation, manual airway clearance, positioning, and tactile stimulation (PROMPT) approach on a preterm neonate with NRDS. The report details the pre-natal, natal, and post-natal history of the patient, including maternal health, pregnancy complications, delivery specifics, initial clinical presentation, and subsequent management. Standard treatments such as exogenous surfactant administration and respiratory support were complemented with PROMPT techniques. The outcomes demonstrate the potential benefits of incorporating physical rehabilitation in the management of NRDS, highlighting improvements in respiratory function and overall clinical stability. This case underscores the importance of multidisciplinary approaches in enhancing the care and prognosis of neonates with NRDS.

## Introduction

Hyaline membrane disease, also referred to as neonatal respiratory distress syndrome (NRDS), is a common and dangerous illness that mostly affects preterm infants. The condition results from a deficiency in pulmonary surfactant synthesis, which is necessary to lower the surface tension in the lungs and avoid alveolar collapse during expiration. A surfactant shortage causes atelectasis, which is the collapse of a lung, as well as poor gas exchange and eventual respiratory failure. Neonates with NRDS are more likely to experience morbidity and mortality, particularly if they are delivered before 37 weeks of gestation [1-4].

The first few hours of life are usually when NRDS manifests clinically. Chest retractions, grunting, nasal flaring, tachypnea (rapid breathing), and cyanosis (bluish coloring of the skin owing to lack of oxygen) are among the symptoms. These symptoms show that the newborn is having difficulty breathing and maintaining enough oxygen [5,6]. Clinical evaluation and imaging techniques, such as a chest X-ray with a distinctive ground-glass look and air bronchograms, are frequently used to confirm the diagnosis.

To lessen the intensity of symptoms and enhance lung function, exogenous surfactant is administered as part of the standard treatment protocol for non-reversible dysplasia of the lungs (NRDS). To ensure appropriate oxygenation and ventilation, respiratory support techniques including mechanical ventilation and continuous positive airway pressure (CPAP) are frequently used. Some newborns endure severe respiratory difficulties even after these procedures, necessitating the use of additional therapeutic approaches to improve outcomes [7,8]. A variety of therapies are included in the physical rehabilitation on oro-motor stimulation, manual airway clearance, positioning, and tactile stimulation (PROMPT) strategy, which aims to improve respiratory function and general physiological stability [9,10]. This case study explores the use of the PROMPT strategy in a neonate with NRDS diagnosis, offering a thorough summary of the neonate's prenatal, natal, and postnatal history in addition to the particular rehabilitation methods used and their effects on the neonate's clinical results.

## Case presentation

Prenatal history

A gravida 1 para 0, a 28-year-old female, who had no significant medical history had routine prenatal checkups and a spontaneous conception. The mother's gestational diabetes was discovered at 28 weeks of pregnancy, and it was controlled with diet. Infections, preeclampsia, or hypertension were not present in the past. Prior to the 30th week, routine ultrasounds revealed decreasing amniotic fluid, or oligohydramnios. The fetal growth was in line with the gestational age. The mother had never smoked, drunk alcohol, or used illegal drugs. She was taking prenatal vitamins, iron, and folic acid supplements.

Natal history

The infant was delivered at 34 weeks of gestation. Due to decreased fetal movements and oligohydramnios, an emergency cesarean section was performed. The APGAR scores were 4 and 6 at 1 and 5 minutes, respectively, indicating moderate distress. The neonate weighed 1800 grams, appropriate for gestational age.

Post-natal history

**Figure 1:**
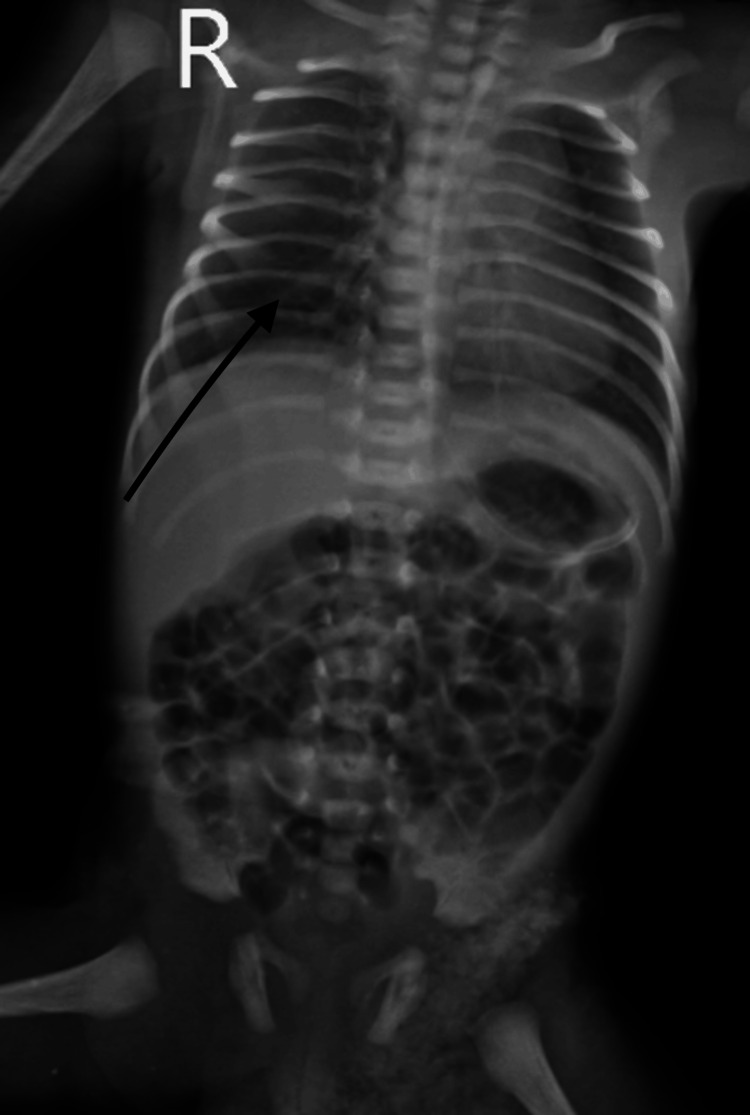
Chest X-ray X-ray showing diffuse, bilateral, and symmetrical granular opacities with bell-shaped thorax and air bronchograms is evident

The neonate exhibited signs of respiratory distress immediately after birth, including tachypnea, grunting, nasal flaring, and retractions. Chest X-ray revealed ground-glass opacities and air bronchograms mentioned in Figure 1 consistent with NRDS. Blood gas analysis showed hypoxemia and respiratory acidosis.

On examination

Before assessment informed consent and assent were taken from the mother. Chest auscultation revealed bilateral coarse breath sounds, consistent with NRDS. The infant was promptly initiated on non-invasive respiratory support with CPAP and received exogenous surfactant therapy as per unit protocol, survival reflexes were mentioned in Table 1.

**Table 1:** Survival reflex

Survival reflexes	
Sucking reflex	Absent
Rooting reflex	Absent
Palmar reflex	Present
Plantar reflex	Present

Further evaluation, including blood gas analysis and by Silverman Anderson respiratory severity score (RSS) confirmed the diagnosis of NRDS, characterized by diffuse bilateral pulmonary infiltrates and a reduced lung volume. Additionally, laboratory investigations were unremarkable, ruling out sepsis or metabolic abnormalities as contributing factors to respiratory distress. The subsequent course of the neonate hospitalization and response to the PROMPT protocol were discussed in Table 2 and treatment went up to the discharge (36 days) of the neonate from NICU.

**Table 2:** PROMPT protocol PROMPT: physical rehabilitation on oro-motor stimulation, manual airway clearance, positioning, and tactile stimulation

Intervention	Intervention	Procedure	Duration
Oromotor stimulation	Cheek stretch	Using your index finger, gently press on the tissue at the nasal bridge. Using your finger, make a C shape and slide it down to the lip's corner after initially going toward the ear. On the opposite side, carry out the same procedure.	2 minutes
	Upper lip stimulation	To compress the tissue, lightly push the index finger on the top lip's corner. Your finger should go from one corner to the center and back again in a circular pattern. To finish the movement, reverse the direction.	2 minutes
	Lower lip stimulation	To compress the skin, lightly press your index finger on the lower lip's corner. Make a circular movement with your finger, beginning at the corner, moving to the middle, and ending at the opposite corner.	2 minutes
	Upper gum stimulation	By starting your finger in the middle of the gum, moving it to the rear of the mouth, and then back to the center, you may gradually apply strong pressure.	2 minutes
	Lower gum stimulation	Place your finger in the middle of the gum, push firmly and steadily, and then gently slide it in the direction of the rear of your mouth.	2 minutes
	Tongue stimulation	Align your finger with the molars by sandwiching it between the side of the tongue and the lower gum. As you move your finger toward the midline, press down to force the tongue in the other way. Press firmly for three seconds on the hard palate after quickly creating a squeezing feeling with your finger against the face. At last, apply pressure with your fingertip until it touches the center of the tongue.	2 minutes
	TMJ Stroking	Temporomandibular joint (TMJ) strokes should be included in oro-motor stimulation using methods that target the muscles surrounding the jaw joint. These techniques involve focusing pressure on the surrounding muscles, kneading the jaw joint softly, and moving in circles. Enhancing orofacial muscular coordination, reducing muscle tension, and increasing blood flow are the goals.	2 minutes
Airway clearance technique	Percussion	To create vibrations in the lung airways, chest percussion entails tapping the chest using percussor cups, which resemble suction cups. By loosening mucus, these vibrations facilitate better cough expulsion.	30 percussion for 5 sets
	Active Gentle Vibrations	During exhale, a short, delicate trill-like motion was used to delicately impart vibrations. After percussion, gentle vibrations were applied to help move secretions toward the bigger airways. The targeted location on the chest wall was covered with the fingers of one hand to manually apply the chest vibrations to each infant. The hand and forearm muscles contracted isometrically to produce a faint vibrating motion. Throughout the procedure, the infant's head was supported by the other hand, which was held with the palm cupped to cradle it.	30 vibrations for 5 sets
	Postural drainage	During the draining of the anterior parts of the left and right upper lobes, the infant was placed flat on the back. The neonate was tilted forward at a ninety-degree angle to allow for the draining of the left and right lateral basal portions of the lower lobes. After that, the top regions of the lower ribs were percussionated. Furthermore, the sides of the chest below the clavicles were pounded, extending into the nipple region, with caution to prevent direct pressure on the sternum.	Each drainage posture was maintained for a period of 5 minutes per position.
Positioning	Swaddling	It is traditional to swaddle newborns, limiting their movement by enveloping them securely in a blanket or piece of fabric. It's a typical technique to help newborns feel safe and sleep better. To reduce hazards, it's essential to swaddle safely, though. The following are some fundamental ideas about the swaddling position: Reverse Placement Permission at the Hips: Put the hands up or face down and skewed legs.	3 hours per day
Tactile stimulations		The baby was stimulated tactilely by being softly caressed with moderate pressure, which required the use of both hands. The infant was placed in the prone (face-down) position during tactile stimulation.	Each stimulation treatment lasted 10 minutes, and they were conducted twice a day, at least 2 hours apart and at least 30 minutes after eating.
Kinesthetic stimulations		The infant received modest movements to provide kinesthetic stimulation to the hip, knee, ankle, shoulder, elbow, and wrist. The baby was placed supine in a crib or on the parent's lap during this phase.	The duration of each stimulation session was 10 minutes, and they were conducted twice a day, at least 2 hours apart and at least 30 minutes after eating.

Stimulation

**Figure 2:**
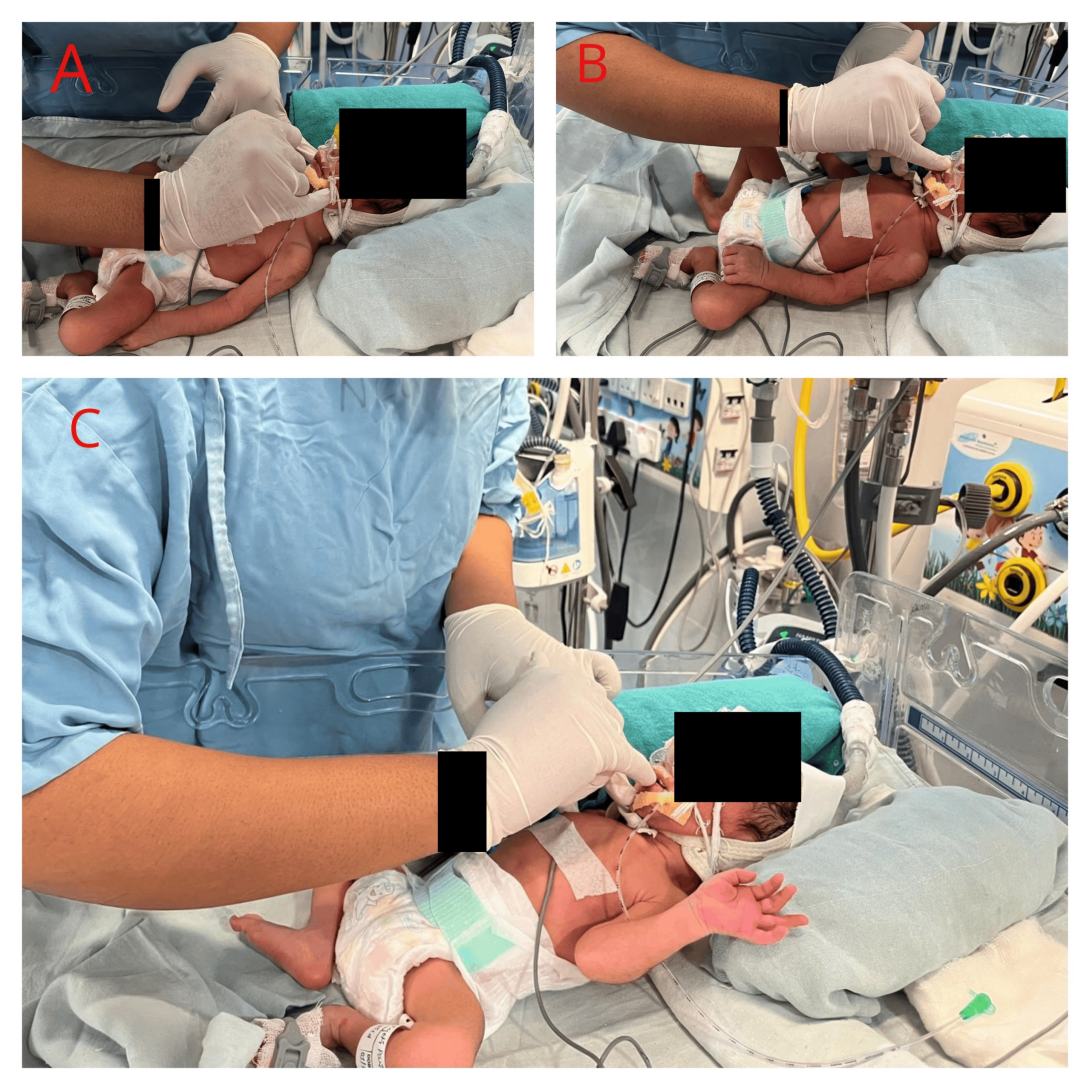
Oro-motor stimulation A: cheek stretch; B: upper lip stimulation; C: lower lip stimulation

For cheek stretch, use your index finger to gently press on the tissue at the nasal bridge, make a C shape, and slide it down to the lip's corner, then repeat on the opposite side. For upper lip stimulation, lightly press the index finger on the corner of the top lip, moving it in a circular pattern from one corner to the center and back. Lower lip stimulation involves lightly pressing the index finger on the lower lip's corner, making a circular movement from the corner to the middle and to the opposite corner. For upper gum stimulation, start your finger in the middle of the gum, and apply gradual pressure while moving to the back of the mouth and then back to the center. Lower gum stimulation involves placing your finger in the middle of the gum, pushing firmly and steadily, and sliding it toward the back of the mouth. Tongue stimulation requires aligning your finger with the molars, pressing down to move the tongue in the opposite direction, pressing firmly for three seconds on the hard palate, creating a squeezing feeling, and then applying pressure with your fingertip to the center of the tongue. Temporomandibular joint (TMJ) stroking incorporates methods that target the muscles around the jaw joint, focusing pressure on the surrounding muscles, kneading the jaw joint softly, and moving in circles to enhance orofacial muscular coordination, reducing muscle tension, and increasing blood flow as shown in Figure 2.

Airway clearance technique

**Figure 3:**
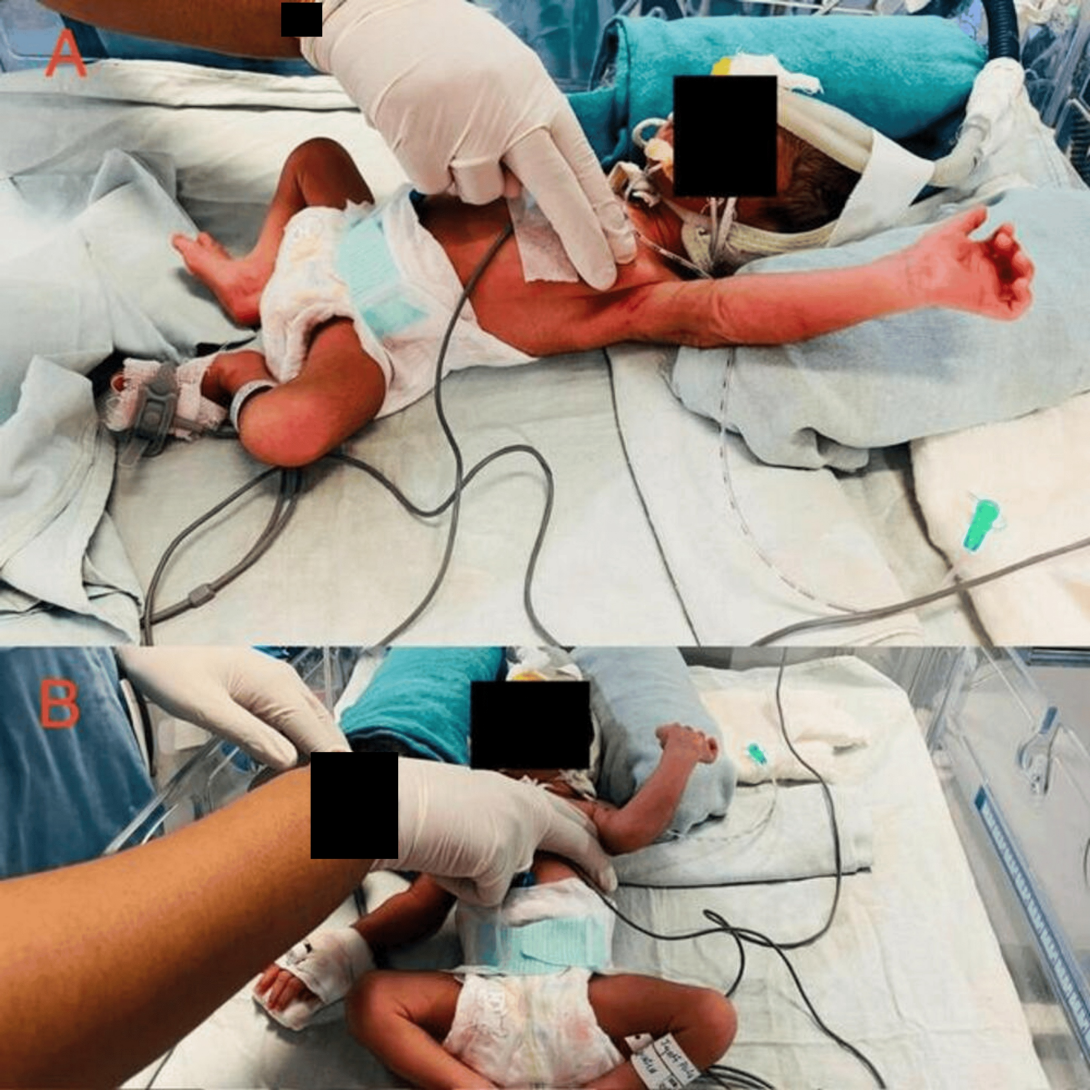
Manual airway clearance technique A: chest percussion; B: chest vibration

Chest percussion involves tapping the chest with percussor cups, resembling suction cups, to create vibrations in the lung airways, loosening mucus for better cough expulsion, with a regimen of 30 percussions for five sets. During exhale, gentle vibrations, applied through a short, delicate trill-like motion, help move secretions toward larger airways. The fingers of one hand manually apply the vibrations to each infant's chest, while the other hand supports the infant's head, with a regimen of 30 vibrations for five sets. Postural drainage involves placing the infant flat on the back to drain the anterior parts of the upper lobes, tilting them forward at a 90-degree angle to drain the lateral basal portions of the lower lobes. The top regions of the lower ribs are then percussed, and the sides of the chest below the clavicles are gently pounded, extending into the nipple region, with caution to avoid direct pressure on the sternum, maintaining each posture for five minutes per position which is shown in Figure 3.

Outcome measures

The intervention went up to the hospital discharge from the NICU; the progress of the neonate was mentioned in Table 3, which included a pre-intervention score and post-intervention score comparison.

**Table 3:** Pre- and post-intervention outcome measures APGAR: appearance, pulse, grimace, activity, respiration; RSS: respiratory severity score

Outcome measure	Pre-intervention	Post-intervention
APGAR	4/10 (1 minute), 6/10 (5 minutes)	10/10
Oral motor assessment scale	10/36	32/36
Silverman Anderson respiratory severity score RSS questionnaire	7	
Length of hospital stay	36 days	

## Discussion

The impact of physical rehabilitation techniques, particularly those involving oro-motor stimulation, manual airway clearance, positioning, and tactile stimulation (collectively referred to as PROMPT), on neonates with RDS has garnered increasing attention in recent years. RDS, a common condition in premature infants, is characterized by insufficient surfactant production leading to lung collapse and impaired gas exchange. This case study explores the efficacy of PROMPT interventions on neonates with RDS, comparing its findings with those from previously published articles on similar interventions.

Oro-motor stimulation involves gentle exercises and stimulation of the oral muscles to enhance feeding skills and respiratory function. This technique is vital for neonates with RDS as it can improve their ability to suck, swallow, and breathe simultaneously, which is crucial for their overall development and health. Studies such as those by Fucile et al. (2011) have demonstrated that oro-motor interventions can significantly enhance feeding efficiency and reduce the duration of hospitalization in preterm infants. The current case study corroborates these findings, indicating that consistent oro-motor stimulation leads to improved oral feeding capabilities and overall respiratory stability [11-14].

Manual airway clearance techniques, including chest physiotherapy and suctioning, aim to maintain airway patency and prevent respiratory complications. Research, such as the work by Knight et al. (2001), has shown that these techniques can be beneficial in managing secretions and reducing the incidence of atelectasis in neonates with RDS. The case study under discussion highlights similar positive outcomes, with infants receiving manual airway clearance showing fewer respiratory complications and better oxygenation levels [15-17].

Positioning strategies, such as prone or lateral positioning, are employed to optimize lung mechanics and improve gas exchange. Several studies, including those by Rivas-Fernandez et al. (2016), have reported that prone positioning can enhance oxygenation and reduce the work of breathing in preterm infants with RDS. The current case study aligns with these findings, demonstrating that strategic positioning can lead to improved respiratory parameters and reduced need for mechanical ventilation.

Tactile stimulation involves gentle touch and massage to promote physiological stability and developmental outcomes. Research by Field (2010) suggests that tactile stimulation can positively affect respiratory rate, heart rate, and overall stress levels in preterm infants. The case study echoes these results, indicating that neonates receiving regular tactile stimulation exhibited improved respiratory function and greater overall stability [18-20].

One key limitation is the delicate condition of these infants, making it challenging to implement intensive rehabilitation without risking additional stress or injury. Additionally, the variability in each neonate’s response to rehabilitation interventions can complicate the standardization of protocols, necessitating highly individualized care plans. This variability can also impact the measurement of outcomes, making it difficult to draw definitive conclusions about the efficacy of specific interventions. Despite these challenges, early rehabilitation is crucial for neonates with RDS. Early intervention, such as the PROMPT approach, which combines oro-motor stimulation, airway clearance, positioning, and tactile stimulation can significantly enhance respiratory function, feeding, and overall stability. By addressing these critical areas early on, rehabilitation can promote better long-term outcomes, reducing the risk of chronic respiratory issues and improving developmental trajectories. Early rehabilitation also supports the critical periods of neuroplasticity in neonates, facilitating more effective and lasting improvements in their health and development.

The findings of the case study are consistent with the broader body of literature on the impact of PROMPT interventions on neonates with RDS. The consensus across various studies is that these rehabilitation techniques contribute to better respiratory outcomes, shorter hospital stays, and enhanced overall development in preterm infants. However, it is important to note that the effectiveness of these interventions can vary based on individual patient characteristics, the timing of intervention, and the specific techniques used. In summary, the case study reinforces the growing body of evidence supporting the use of oro-motor stimulation, manual airway clearance, positioning, and tactile stimulation in the management of neonates with RDS. These interventions collectively enhance respiratory function, promote developmental outcomes, and reduce the burden of respiratory complications, thereby offering a comprehensive approach to neonatal care. Further research and larger clinical trials are warranted to refine these techniques and establish standardized protocols for their implementation in neonatal intensive care units (NICUs).

## Conclusions

This case study suggests PROMPT, a physical rehabilitation approach combining oro-motor stimulation, airway clearance, positioning, and tactile stimulation, improves respiratory function, feeding, and stability in neonates with RDS. This aligns with prior research on each PROMPT component's benefits, which suggests a comprehensive approach to managing RDS. While promising, further studies are needed to refine techniques, establish protocols, and confirm long-term effects. Overall, the case study highlights the potential of physical rehabilitation as a valuable addition to neonatal care for infants with RDS.
